# Comparisons of Clinical Outcomes and Prognoses in Patients With Gastroesophageal Junction Adenocarcinoma, by Transthoracic and Transabdominal Hiatal Approaches: A Teaching Hospital Retrospective Cohort Study

**DOI:** 10.1097/MD.0000000000002277

**Published:** 2015-12-18

**Authors:** Jinzhe Zhou, Hao Wang, Zhaojian Niu, Dong Chen, Dongsheng Wang, Liang Lv, Yu Li, Jian Zhang, Shougen Cao, Yi Shen, Yanbing Zhou

**Affiliations:** Department of General Surgery, Tongji Hospital, Tongji University, Shanghai (JZ); The People's Hospital of Dongying City, Shan Dong Province (HW); and Affiliated Hospital of Qingdao University, Qingdao, China (ZN, DC, DW, LL, YL, JZ, SC, YS, YZ).

## Abstract

To compare the clinical outcomes and prognoses in patients with gastroesophageal junction adenocarcinoma (Siewert type II/III), by transthoracic and transabdominal hiatal approaches.

Siewert II/III gastroesophageal junction adenocarcinomas patients (334 cases) underwent different surgical procedures at the Affiliated Hospital of Qingdao University from July 2007 to July 2012 and were analyzed retrospectively. In total, 140 patients underwent surgery by the transthoracic approach, and 194 patients underwent the transabdominal hiatal approach mainly with radical total and proximal gastrectomy (D2). All patients were followed up by telephone review or by outpatient reexamination until July 2013. The surgically related and clinical outcomes were compared using the χ^2^ test, *t* test, Fisher exact test, or nonparametric rank sum test according to different data. The survival curve was drawn by the Kaplan–Meier method and survival analysis used Cox regression analysis.

The operative time, length of resected esophagus, number of lymph nodes harvested, postoperative pain scores, postoperative hospital stay, time of antibiotics use, postoperative morbidity, and costs for the transabdominal surgery group were better than that of the transthoracic group. The overall 5-year survival rate was 35.3% and 40.3%, respectively, in the transthoracic and transabdominal surgery groups, and differences were not statistically significant (x^2^ = 2.311, *P* > 0.05). The hazard ratio of death for the transthoracic compared with the transabdominal approach was 1.27 (0.93–1.72, *P* > 0.05). According to tumor node metastasis (TNM) staging, stratification analysis showed that stage III patient overall survival rates were 25.7% and 37.2%, respectively. The differences were statistically significant (x^2^ = 4.127, *P* < 0.05). In uni- and multivariate Cox regression analysis, the hazard ratio for the transabdominal versus the transthoracic approach was 0.66 (0 43 to 0.99, *P* < 0.05) and 1.47 (1.05–2.06, *P* < 0.05), respectively.

There were no significant differences of 5-year overall survival in TNM stage I and II of the Siewert II/III adenocarcinoma patients, but improved survival of TNM stage III patients undergoing transabdominal hiatal compared with transthoracic total radical and proximal gastrectomy. The short-term clinical outcomes improved with the transabdominal hiatial surgery group.

## INTRODUCTION

Despite a decreasing global incidence of distal gastric cancer, the frequency of gastroesophageal junction (GEJ) adenocarcinoma has greatly increased in the developed countries, especially in Western countries.^1,2^ In eastern Asian countries, including Japan and Korea, most tumors in the GEJ are reported to be Siewert type II and III,^3,4^ which is consistent with our center.

Owing to its poor prognosis, GEJ adenocarcinoma has received increasing attention in recent years, and surgery provides the best chance for cure. According to the widely accepted Siewert system, based on the epicenter of the GEJ, GEJ adenocarcinomas are usually classified into 3 categories,^5^ with different surgical treatment approaches used to improve survival. The left thoracoabdominal approach is used to treat Siewert type I squamous cell carcinoma, with wide excision of the tumor and peritumoral tissues, and extended lymph node dissection in the mediastinum and abdomen. Controversy, however, exists regarding the choice of thoracotomy or the transabdominal hiatal approach for treatment of Siewert type II and III GEJ adenocarcinoma.^6^ In our study, we retrospectively analyzed data on 334 patients with Siewert II and III GEJ adenocarcinoma who underwent surgery at the Affiliated Hospital of Qingdao University between July 2007 and July 2012. Our aim was to evaluate the short-term and the long-term outcomes of the transthoracic versus transabdominal hiatal approaches.

## MATERIALS AND METHODS

### The Clinicopathologic Characteristics of Gastroesophageal Junction Adenocarcinoma Patients in the 2 Surgery Groups

In the gastric cancer database, data were collected retrospectively, for all 334 patients with histologically confirmed adenocarcinoma of Siewert type II and III who had undergone a curative surgery. The study included 282 male and 52 female patients with ages ranging from 31 years to 88 years of age, and with a mean age of 64 years. All patients were diagnosed by preoperative biopsy and postoperative pathology. Preoperative evaluations routinely included upper gastrointestinal endoscopy, computed tomography scanning, barium swallow measurements, or endoscopic ultrasonography. In selected patients, laboratory indicators, including blood routing, biochemical examinations, tumor markers, and cardiorespiratory or other surgical data, which would influence organ function were included. In the transthoracic group, there were 140 patients that received total transthoracic or proximal gastrectomy, and D2 lymph node dissection, whereas in the transabdominal hiatal group, 194 patients received total transabdominal hiatal or proximal gastrectomy, and D2 lymph node dissection. As is shown in Table 1, the clinical characteristics, pathology data, and preoperative comorbidities showed no differences between the 2 groups (*P* > 0.05).

**TABLE 1:**
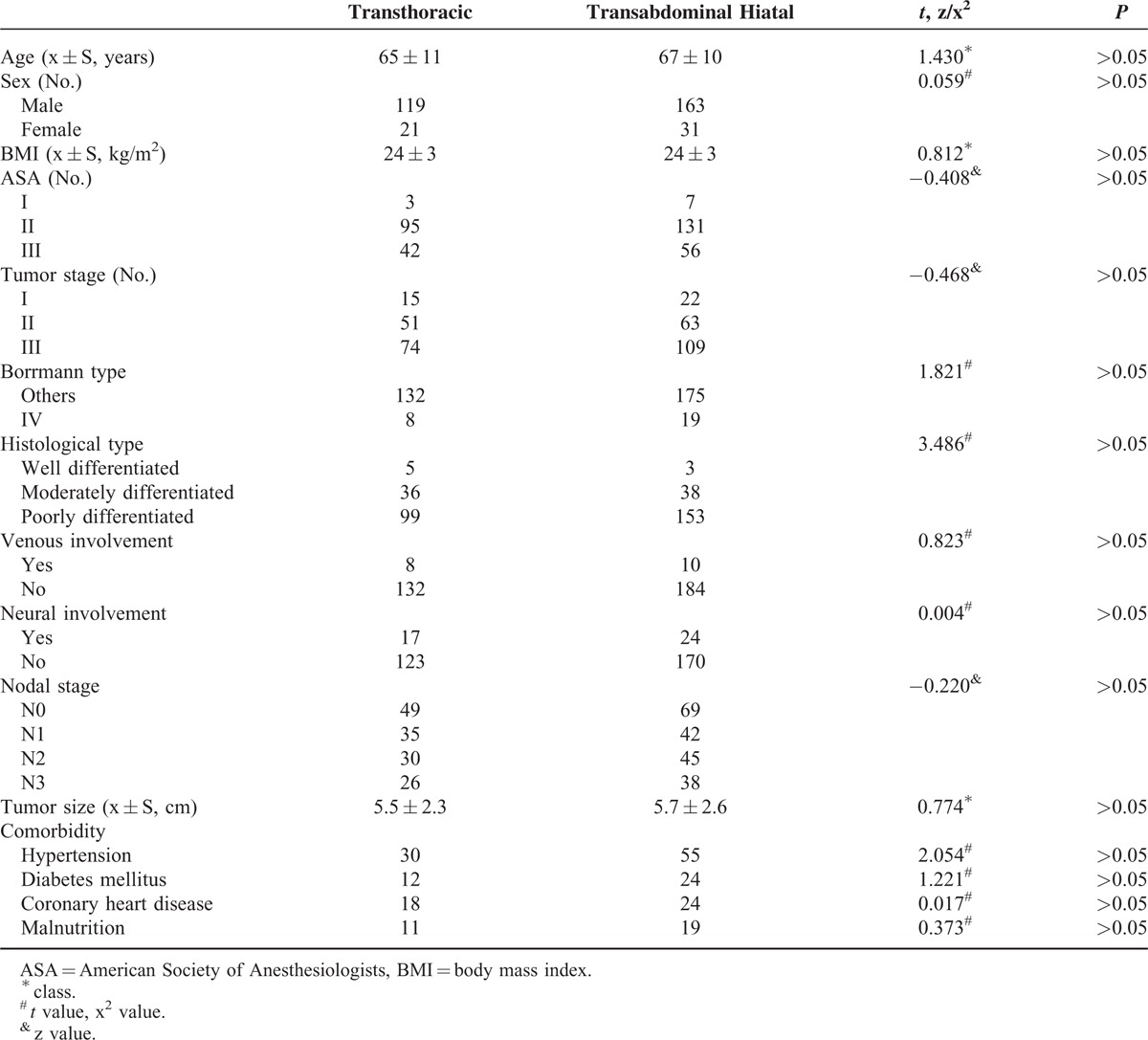
The Clinicopathologic Characteristics of Gastroesophageal Junction Adenocarcinoma Patients in the Transabdominal Hiatal Group and Transthoracic Group

Postoperative adjuvant therapy was usually capecitabine plus oxaliplatin for TNM stage II/III GEJ adenocarcinomas.

### Inclusion and Exclusion Criteria

All the eligible Siewert II/III GEJ adenocarcinoma patients were referred to general or thoracic surgeon for further surgery by primary gastroenterologist after getting the informed consent and with full respect for the different surgeon selection of patients and their families. The inclusion criteria were as follows: all the patients had a histologically proven adenocarcinoma and were identified as Siewert type II and III, which was centered within 1 cm above to 5 cm below the GEJ, as assessed by preoperative gastroscopy. Intraoperative results, biopsies, and postoperative pathologic findings with no distant metastasis were also included. The exclusion criteria were: squamous cell carcinoma and Siewert type I, which was located 1 to 5 cm above the GEJ, preoperative neoadjuvant chemotherapy or radiation therapy, previous palliative operation, and exploratory laparotomy and laparoscopic or thoracoscopy approaches.

### Surgical Procedures

All the surgeries were performed under general anesthesia or combined general and epidural anesthesia by experienced surgeons who had previously performed more than 50 radical gastrectomies with D2 dissection annually. Meanwhile, the extent of lymph node dissection and anastomosis reached homogeneity under the same anatomy and surgical principle.^7,8^ Cytologic examination of abdominal washings with 100 mL of physiologic saline solution (37 °C) collected from the left subphrenic area or the pouch of Douglas was performed at the beginning of the operation. Combined organ resection was performed in the case of a tumor invading adjacent organs without distant metastasis, according to the patient's general condition.

In the transthoracic group, an incision was made in the seventh left intercostal space. According to the anatomy of the lower esophagus and the stomach, total or proximal gastrectomy through the esophageal hiatus was performed, and the type of digestive tract reconstruction was esophagogastrostomy or Roux-en-Y esophagojejunostomy. Lymphadenectomy mainly involved the lower thoracic paraesophageal (No. 110), supradiaphragmatic (No. 111), infradiaphragmatic (No. 19), lymph nodes in the esophageal hiatus of the diaphragm (No. 20), and perigastric lymph nodes (No. 3, 5, 2, 4, 7, 9, and 11p). In the transabdominal hiatal group, total or proximal gastrectomy was performed through an upper abdomen midline incision made by the general surgeon. The type of anastomosis was the same as in the transthoracic group. The regional lymph node dissection included No. 1, 3, 5, 2, 4, 6, 7, 8a, 9, 11p, 11d, No. 19, and No. 20, and 33 patients underwent extended lymph node dissection at the splenic hilium (No. 10).

### Follow-Up

All patients were visited at the outpatient clinic or by telephone follow-up or by other information. The quality-of-life indicators, including dietary intake and reflux information, tumor markers, and imaging data (endoscopy, computed tomography) were the main postoperative evaluation indicators.^7^ Follow-up time was calculated from the day of operation to the end of the follow-up study (July 2013) or until death.

### Statistical Analysis

Statistical analyses were performed using SPSS version 18.0 (SPSS, Chicago, IL). For the clinical data, Student *t* test or the Mann–Whitney *U* test was used for continuous data. χ^2^ or Fisher exact tests were used to compare categorical data. The nonparametric Kruskal–Wallis rank test was used for ranked data. The survival curve was drawn by the Kaplan–Meier method, and survivals between groups were compared using the log-rank test and the Cox regression analysis. A *P* value <0.05 was considered statistically significant throughout the study.

## RESULTS

### Clinical Outcome of 2 Groups After Operations

Curative (R0) resection was performed in all the patients in the 2 groups. The results showed there were no patient deaths postoperatively within 30 days of surgery. The operation time and length of hospital stay after operation was shorter, and the incidence of overall morbidity was lower in the transabdominal group compared with those in the transthoracic group (*P* < 0.05). There were no statistically significant differences in postoperative complications between the 2 groups (*P* > 0.05) except with pulmonary infections and anastomotic leakage (*P* < 0.05). Other indicators of combined organ resection, length of esophageal resection, total number of lymph nodes obtained, postoperative pain score, the duration of antibiotic treatment, hospitalization, expenses, and Clavien–Dindo classification^9^ of postoperative complications showed statistically significant differences between the 2 groups (*P* < 0.05; Table 2).

**TABLE 2:**
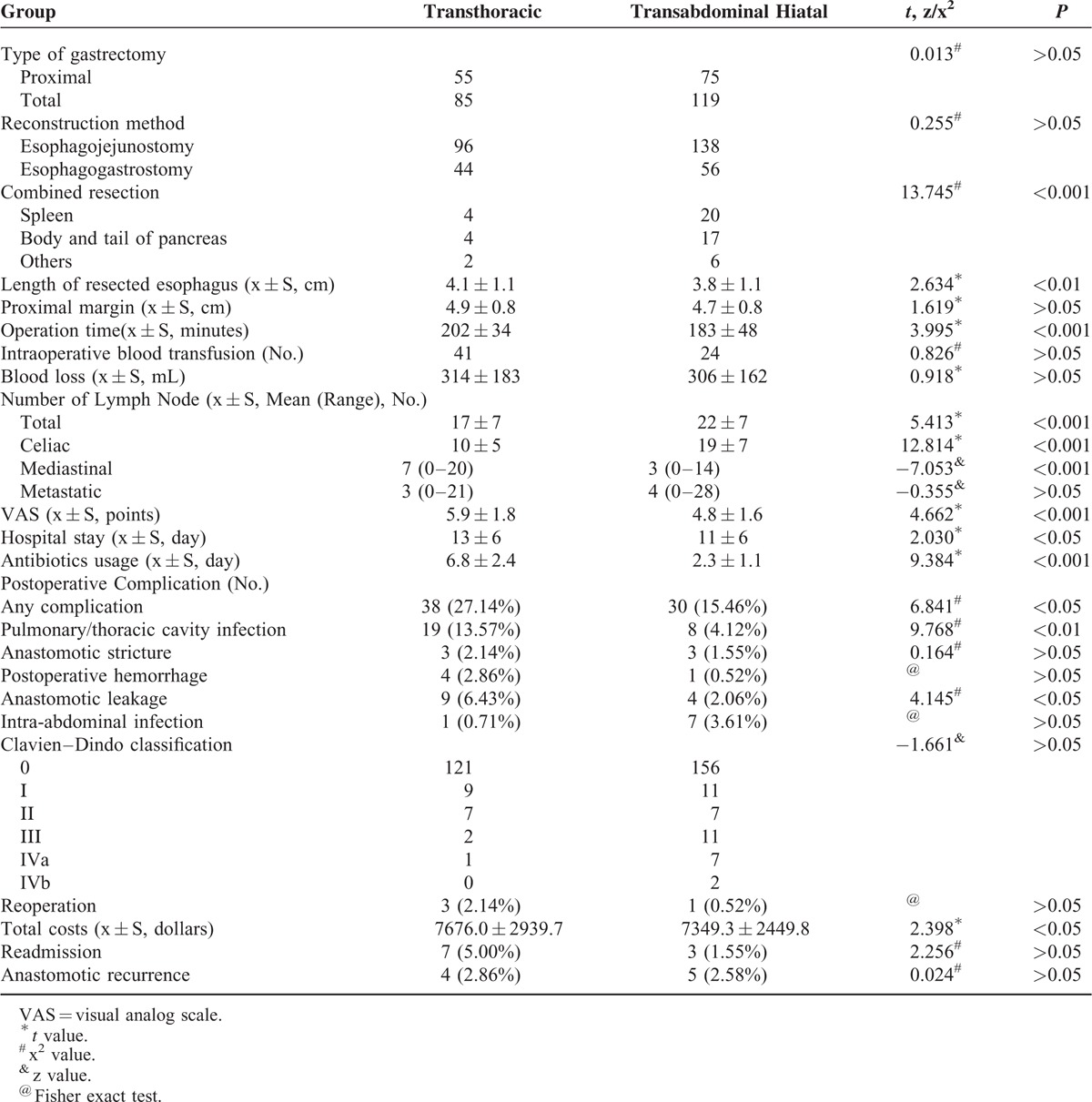
The Clinical Outcomes of Gastroesophageal Junction Adenocarcinoma Patients in the, Transabdominal Hiatal Group and Transthoracic Group

### Prognosis of the 2 Groups

**FIGURE 1:**
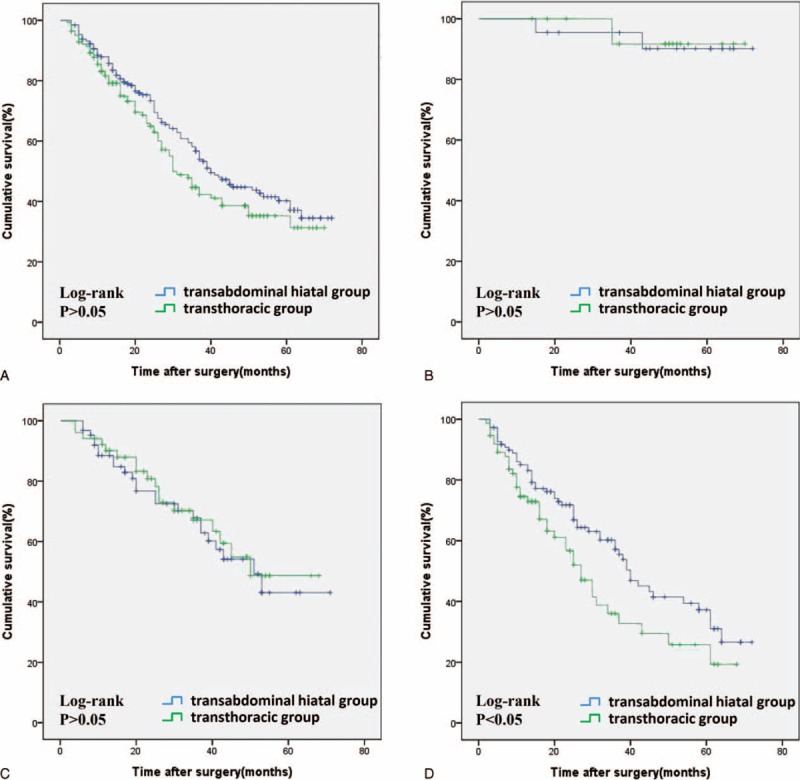
Postoperative survival curve of the transabdominal hiatal and transthoracic procedures. A, Overall survival curve. B, Tumor node metastasis stage І survival curve. C, Tumor node metastasis stage ІІ survival curve. D, Tumor node metastasis stage ІІІ survival curve, transabdominal hiatal group transthoracic group.

Follow-up was obtained in 302 of 334 (90.42%) patients with a median follow-up of 38 months (range: 2–72 months). For the transabdominal hiatal and transthoracic procedures, the rate of overall survival at 5 years was 40.3% and 35.3%, respectively, and there were no significant differences between the 2 groups (*P* > 0.05; Figure 1A). The HR of death for the transthoracic compared with the transabdominal approach was 1.27 (0.93 to 1.72, *P* > 0.05). In subgroup analysis based on the TNM classification according to the International Union Against Cancer/American Joint Committee on Cancer TNM Classification for Gastric Carcinoma (seventh edition),^10^ although there were no statistically significant differences in 5-year overall survival of TNM stage I and II between the 2 groups [91.7% and 48.7% in the transthoracic group, and 90.2% and 43.1% in the transabdominal group, respectively, *P* > 0.05, Figure 1B and C, hazard ratio (HR) was 0.87 (0.11 to 13.40) and 1.11 (1.61 to 2.06), respectively, *P* > 0.05], there was a clear trend toward improved survival of TNM stage III patients with the transabdominal approach [25.7% versus 37.2%, *P* < 0.05, Figure 1D, and HR was 0.66 (0 43 to 0.99, *P* < 0.05)]. In multivariate Cox regression analysis with stepwise selection, variables included age, sex, tumor size, tumor site, histologic type, Borrmann type, TNM stage were analyzed, TNM stage III remained significantly associated with survival of patients, and HR was 1.47 (1.05 to 2.06, *P* < 0.05).

## DISCUSSION

Gastroesophageal junction adenocarcinomas were defined as tumors whose centers were within 5 cm proximal and distal of the GEJ, and were classified into 3 subtypes according to the Siewert classification, which was described as follows: Siewert type I (distal esophageal adenocarcinoma) was a tumor with the epicenter located 1 to 5 cm above GEJ regardless of invasion to GEJ, Siewert type II (true carcinoma of the cardia) was a tumor invading GEJ with the epicenter located between 1 cm above and 2 cm below GEJ, and Siewert type III (carcinoma of the subcardia) was a tumor invading GEJ with the epicenter located 2 to 5 cm below the GEJ.^5,11^ Regarding Siewert type II and III tumors, the classification or treatment approaches, however, still remain controversial.^12^ Because of the unusual location, the tumors are biologically aggressive and they usually lead to a low survival rate.

Until recently, surgery was still the most effective curative treatment for GEJ adenocarcinoma, but the surgical approach was quite different because of the unusual anatomic site. The 3 most widely used approaches involve left thoracoabdominal, transthoracic, and the transabdominal hiatal approach. The advantage of the left thoracoabdominal approach is the convenience for abdominal and mediastinal lymph node dissection, but it may cause extensive trauma and could increase the incidence of postoperative complications. By the transthoracic approach, a thorough mediastinal nodal dissection is undertaken with sufficient length of the oesophagectomy to use for constructing the esophageal anastomosis. This approach is generally performed to treat Siewert type I cases in Europe and America. The operative approaches for Siewert type I and II adenocarcinomas still remain controversial. A Dutch randomized controlled trial comparing the right transthoracic and transabdominal hiatal approaches for Siewert type I and II tumors found postoperative morbidity was higher with the transthoracic approach, although there were no statistically significant differences in overall survival in the entire study population between the 2 groups. There was, however, a clear trend toward improved survival with the transthoracic approach, and the transthoracic approach via a right thoracotomy was recommended for Siewert type I tumors, but not for Siewert type II or III tumors.^13,14^ Sasako et al^15^ demonstrated that there was no survival benefit in the postoperative overall survival, and perioperative morbidity was higher with the transthoracic approach when compared with the transabdominal hiatal approach in a phase III randomized controlled trial for mainly Siewert type II and III tumors. The transthoracic approach also aggravated weight loss symptoms, and respiratory functions compared with the transabdominal hiatal approach.^16^

Subgroup analyses showed no survival benefit for Siewert type II patients with the transthoracic approach, and the transabdominal hiatal approach was associated with better survival than the transthoracic approach for Siewert type III patients.^14^ In our study, intraoperative index monitoring showed shorter operation time, more numbers of lymph nodes harvested, and the postoperative (30 days) index monitoring showed a lower incidence of morbidity, lower postoperative pain scores, shorter postoperative hospital stay, and shorter time of antibiotic treatment in the transabdominal hiatal group than in the transthoracic group. There were no benefits in improving the 5-year overall survival for TNM stage I and II cases, but improved survival was observed for TNM stage III cases with the transabdominal hiatal approach, we think this may mainly relate to lymph node metastasis and radical lymphdetectomy, in our study, the dissection of total number of lymph node has some advantage with the transabdominal hiatal approach than the other. In addition, uni- and multivariate Cox regression analysis showed that earlier stage with better prognosis, the clinical stage had a significant effect on the prognosis of the patients, the key factors to improve prognosis was associated to early detection and surgical approaches.

In an analysis of the advantages of the transabdominal hiatal approach used in this study, the incidence of lymphatic metastasis in GEJ adenocarcinomas toward the mediastinum demonstrated that the perigastric or para-abdominal aorta showed differences. Lymph node metastasis to the area of Siewert type III tumors was the same as metastasis to the upper abdomen. For Siewert type II tumors, the area of lymph node metastasis included both the abdomen and mediastinum mainly toward the peritumor and abdomen. Lymph node metastasis in the area of the lymphatic flow around the tumor was an indicator of poor prognosis. The number of lymph nodes dissected also had an important influence on the prognosis of the patients. Pedrazzani et al^17^ showed that the lymphatic flow of Siewert type II cases was mainly directed toward the abdomen, and lymph node metastasis reflected a high risk to the paracardial and lesser curvature lymph nodes (No. 1, 2, 3, and 7), but a low risk to No. 4sa, 4sb, 4d, 5, and 6 nodes. Para-aortic lymph node (No. 16a2) involvement rate was approximately 15%.^18,19^ Dissection of lower mediastinal lymph nodes is necessary for treatment of all types of GEJ adenocarcinomas because of the high risk of metastasis. The upper or middle mediastinum lymph node metastasis from Siewert type II or III tumors, however, was relatively rare, so it is not necessary to make a thorough dissection of this area via thoracotomy.^20,21^ The transabdominal hiatal approach makes the abdominal lymph node dissection easier, and especially shows a great advantage in lymph node dissection in the area of the superior edge of the pancreas (No. 7, 8, 9, 11p, and 11d) and splenic hilum (No. 10), compared with the transthoracic approach.

## CONCLUSIONS

We conclude that there were no differences in 5-year overall survival rates for TNM stage I and II of Siewert type II and III tumors in patients undergoing total radical or proximal gastrectomy by the transabdominal hiatal approach compared with the transthoracic approach. There, however, was clearly an improved survival of TNM stage III cases using the transabdominal hiatal approach. The transabdominal hiatal approach was less invasive and had a lower postoperative morbidity and more short-term advantages. This was a retrospective study in a single center, and a multicenter and prospective study is necessary in the future.
